# Conserved gammaherpesvirus protein kinase counters myeloid cell-specific type I IFN receptor but not IRF-3 expression to promote the establishment of chronic latent infection in the spleen

**DOI:** 10.1128/jvi.01110-26

**Published:** 2026-09-01

**Authors:** Erika R. Johansen, Matthew A. Brown, Xander G. Bradeen, Cade R. Rahlf, Daniela Avila, Damon. L. Schmalzriedt, Vera L. Tarakanova

**Affiliations:** 1 Department of Microbiology and Immunology, Medical College of Wisconsin 735651, Milwaukee, Wisconsin, USA; 2 Cancer Center, Medical College of Wisconsin, Milwaukee, Wisconsin, USA; University of Virginia, Charlottesville, Virginia, USA

**Keywords:** IRF-3, type I interferon, chronic infection, gammaherpesvirus protein kinase

## Abstract

Gammaherpesviruses are prevalent pathogens that are associated with cancers and autoimmune diseases. All gammaherpesviruses encode a conserved protein kinase. Expression of orf36, the protein kinase encoded by murine gammaherpesvirus 68 (MHV68), supports the establishment of a latent viral reservoir in splenic B cells *in vivo*. Specifically, orf36 antagonizes myeloid cell-intrinsic STAT1 function to support the passage of MHV68 from myeloid cells to splenic B cells. STAT1 is a ubiquitously expressed transcription factor and a critical effector of interferon (IFN) antiviral functions. To define the extent to which the observed orf36-STAT1 antagonism reflected orf36 antagonism of the type I IFN axis, this study employed mouse models of myeloid cell-specific type I IFN receptor (IFNAR1) or interferon regulatory factor 3 (IRF-3) deficiencies. Myeloid cell-specific IFNAR1 deficiency resulted in the rescue of the attenuated splenic latent reservoir of the orf36 null MHV68, similar to that observed upon myeloid cell-specific STAT1 deficiency, refining the viral antagonism of the IFNAR1-STAT1 axis. However, and unlike that observed for IFNAR1, myeloid cell-specific deficiency of IRF-3, the transcription factor that drives type I IFN expression, failed to affect the attenuated chronic infection of the orf36 null MHV68 mutant. Unexpectedly, wild-type MHV68 reactivation in the spleen was attenuated in mice with myeloid cell-specific IRF-3 deficiency. Thus, the current study highlights a myeloid cell-specific antagonism between conserved gammaherpesvirus protein kinase and type I IFN receptor and unveils an intriguing proviral role of myeloid-specific IRF-3 expression during chronic gammaherpesvirus infection.

**IMPORTANCE:**

This study focuses on defining the mechanisms by which conserved gammaherpesvirus protein kinase facilitates establishment of chronic infection *in vivo*. We previously demonstrated that the viral protein kinase antagonizes STAT1 function in myeloid cells to allow gammaherpesvirus passage from myeloid cells to B cells in the spleen. Using a combination of virus and host genetics, this study demonstrates that the previously observed antagonism between viral protein kinase and STAT1 is at least in part due to antagonism of type I interferon (IFN) signaling. Despite the well-established role of IRF-3 in type I IFN expression, myeloid cell-specific IRF-3 deficiency failed to rescue attenuated chronic infection of viral kinase null gammaherpesvirus. Unexpectedly, splenic reactivation of wild-type gammaherpesvirus was decreased in mice with myeloid cell-specific IRF-3 deficiency, unveiling a proviral role of IRF-3 expressed by myeloid cells during chronic gammaherpesvirus infection.

## INTRODUCTION

Gammaherpesviruses are pervasive pathogens that establish life-long infection and are associated with malignancies and autoimmune diseases (1, 2). All gammaherpesviruses encode a conserved protein kinase, with multiple host and viral substrates identified in cultured primary cells or transformed cell lines undergoing lytic infection or reactivation (3–8). However, the physiological relevance of many gammaherpesvirus protein kinase substrates identified *in vitro* has not yet been verified in the context of natural infection. Gammaherpesvirus protein kinase is classically considered a lytic cycle protein, expressed with early-late kinetics. Accordingly, we showed that the expression and enzymatic activity of the murine gammaherpesvirus 68 (MHV68) protein kinase (encoded by *orf36*) facilitate MHV68 replication in primary macrophages *in vitro* (5, 9). Importantly, emerging evidence points to the critical role of the gammaherpesvirus protein kinase during chronic infection that is predominantly associated with the latent life cycle. We and others demonstrated that the expression and enzymatic activity of the MHV68 protein kinase *orf36* facilitate the establishment of the latent viral reservoir and viral reactivation during chronic infection (4, 10, 11).

Type I interferon (IFN) signaling is a critical host defense system, including during gammaherpesvirus infection. Multiple type I IFNs ligate a single ubiquitously expressed type I IFN receptor (IFNAR), with subsequent phosphorylation and activation of transcription factor STAT1 that drives expression of IFN-stimulated genes (ISGs). We showed that interferon regulatory factor 3 (IRF-3) was important for induction of interferon beta (IFNβ) and interferon alpha (IFNα) expression in MHV68-infected primary macrophages *in vitro* (12). Conserved gammaherpesvirus protein kinases, including MHV68 orf36, were reported to associate with IRF-3 and antagonize transcription of IFNβ in fibroblasts (4). In contrast, we found that type I IFN expression was robustly induced in primary macrophages infected with wild-type (WT) MHV68 along with STAT1 phosphorylation and ISG expression (13). STAT1 phosphorylation, messenger RNA (mRNA) levels of IFNα (but not IFNβ), and ISG expression were further increased in primary macrophages infected with the orf36 null MHV68 mutant (N36S) (12). Correspondingly, the attenuated replication of the N36S MHV68 mutant was partially rescued in primary macrophages derived from *Irf3^−/−^* mice or mice lacking type I IFN receptor (*Ifnar1^−/−^*) (12).

The interplay between gammaherpesvirus infection and type I IFN signaling *in vivo* has traditionally been studied using a mouse model of global type I IFN receptor deficiency (*Ifnar1^−/−^*). *Ifnar1^−/−^* mice experience high mortality following MHV68 infection, with a corresponding increase in lytic viral titers that remain significantly elevated in survivors of acute infection for at least 3 weeks post-infection (14, 15). Attenuated replication of the N36S mutant was partially rescued in lungs of *Ifnar1^−/−^* mice at 7 days post-infection, along with partial rescue of N36S DNA levels in the *Ifnar1^−/−^* spleens at 14 days post-infection (4). However, given the protracted high levels of MHV68 lytic replication in *Ifnar1^−/−^* mice, it is unclear whether the observed increase in N36S DNA levels at 14 days post-infection reflected increased lytic replication and/or the size of the latent reservoir.

Similar to that observed in *Ifnar1^−/−^* mice, acute lung titers of WT MHV68 were increased in *Irf3^−/−^* mice (16). In contrast, persistent lung replication and establishment of splenic latent reservoir were attenuated in *Irf3^−/−^* mice infected with WT MHV68, revealing an unexpected proviral role of global IRF-3 expression during chronic infection (16). Unlike that observed for global IFNAR1 deficiency, global IRF-3 deficiency had no effect on the attenuated acute lung replication and establishment of N36S latent reservoir in the spleen (16).

Myeloid cells, such as monocytes, macrophages, and dendritic cells, represent a physiologically relevant cell type for gammaherpesvirus infection (17–23). Macrophages, along with epithelial cells, support acute MHV68 replication in the lungs (24). Importantly, Lysozyme M-expressing cells represent a bottleneck that the virus must pass through in order to establish chronic infection in splenic B cells (25). Lysozyme M is primarily expressed in macrophages, a small subset of dendritic cells, and most granulocytes (26), with only the former two cell populations known to support gammaherpesvirus infection. Therefore, it is possible that MHV68 infection of LysM-expressing macrophages precedes establishment of a latent reservoir in splenic B cells.

Having observed attenuated replication of the N36S mutant in primary macrophage cultures along with increased expression of ISGs and phosphorylation of STAT1 (12), we next wanted to determine the extent to which MHV68 orf36 antagonizes IFN signaling in LysM-expressing cells *in vivo*. Given the very low number of infected myeloid cells, even for WT MHV68, and concurrent expression of both type I and type II IFN during chronic infection, a genetic approach was employed in our previous study (27). Specifically, a LysM-driven Cre recombinase allele was combined with conditional *Stat1* alleles to achieve attenuated STAT1 expression and IFN signaling in myeloid cells. Interestingly, while myeloid-specific STAT1 deficiency resulted in significantly elevated acute and persistent lung replication of WT MHV68, there was no effect on the establishment of the splenic latent reservoir (27). In contrast, the attenuated establishment of the N36S latent reservoir in splenic B cells was rescued in mice with myeloid-specific STAT1 deficiency (27). Thus, using a combination of viral and host genetics, we demonstrated that MHV68 orf36 antagonizes antiviral STAT1 functions in LysM-expressing myeloid cells to promote infection and establishment of the latent reservoir in splenic B cells *in vivo* (27).

STAT1 is a critical effector of all classical interferon (IFN) responses. However, STAT1 can be activated by other cytokines, such as IL-27, which primarily activates STAT1 and STAT3 downstream of its receptor (28). Thus, the observed antagonism between MHV68 orf36 and myeloid-intrinsic STAT1 during the establishment of chronic infection (27) could not be unequivocally ascribed to the antagonism of the IFN-STAT1 signaling axis. To determine the extent to which MHV68 orf36 antagonizes myeloid cell intrinsic type I IFN expression and signaling to facilitate the establishment of chronic latent MHV68 infection, this study employed mouse models of myeloid cell-specific IRF-3 or IFNAR deficiency. In these models, expression of either IRF-3 or IFNAR1 was targeted in myeloid cells by combining *Lyz2* promoter-driven Cre recombinase expression with conditional *Irf3* or *Ifnar1* alleles. We found that myeloid cell-specific IFNAR1 deficiency rescued the attenuated splenic latent reservoir of the orf36 null MHV68 mutant (N36S), similar to that previously observed for myeloid cell-specific STAT1 deficiency (27). In contrast, myeloid cell-specific IRF-3 deficiency failed to rescue the attenuated chronic infection of the N36S MHV68 mutant. Interestingly, myeloid cell-specific IRF-3 deficiency led to attenuated *ex vivo* reactivation of WT MHV68 from splenocytes, identifying myeloid cells as one of the cell types supporting proviral effects of IRF-3 during the establishment of chronic gammaherpesvirus infection.

## RESULTS

### Validation of mouse model of myeloid cell-specific IFNAR1 deficiency

Following intranasal inoculation, MHV68 acutely replicates in epithelial cells and macrophages of the lung (24), with subsequent dissemination to the spleen (29). We previously observed that myeloid-specific STAT1 deficiency (achieved by combining conditional *Stat1* alleles with a *Lyz2* promoter-driven Cre recombinase allele) increases acute replication of WT MHV68 and rescues attenuated splenic latent reservoir of the N36S mutant that lacks orf36 expression (4, 27). In the current study, additional genetic mouse models were employed to determine the extent to which the previously observed acute and chronic infection phenotypes were due to myeloid cell-intrinsic type I IFN expression and signaling. To test the role of myeloid cell-intrinsic IRF-3 expression, we obtained a genetic mouse model published by the Taniguchi group where *Irf3* conditional alleles (30) were combined with *Lyz2* promoter-driven Cre recombinase (26, 30). To test the role of myeloid cell-intrinsic type I IFN signaling, we generated a new mouse model where the *Lyz2* promoter-driven Cre recombinase allele was combined with conditional *Ifnar1* alleles (31).

**Fig 1:**
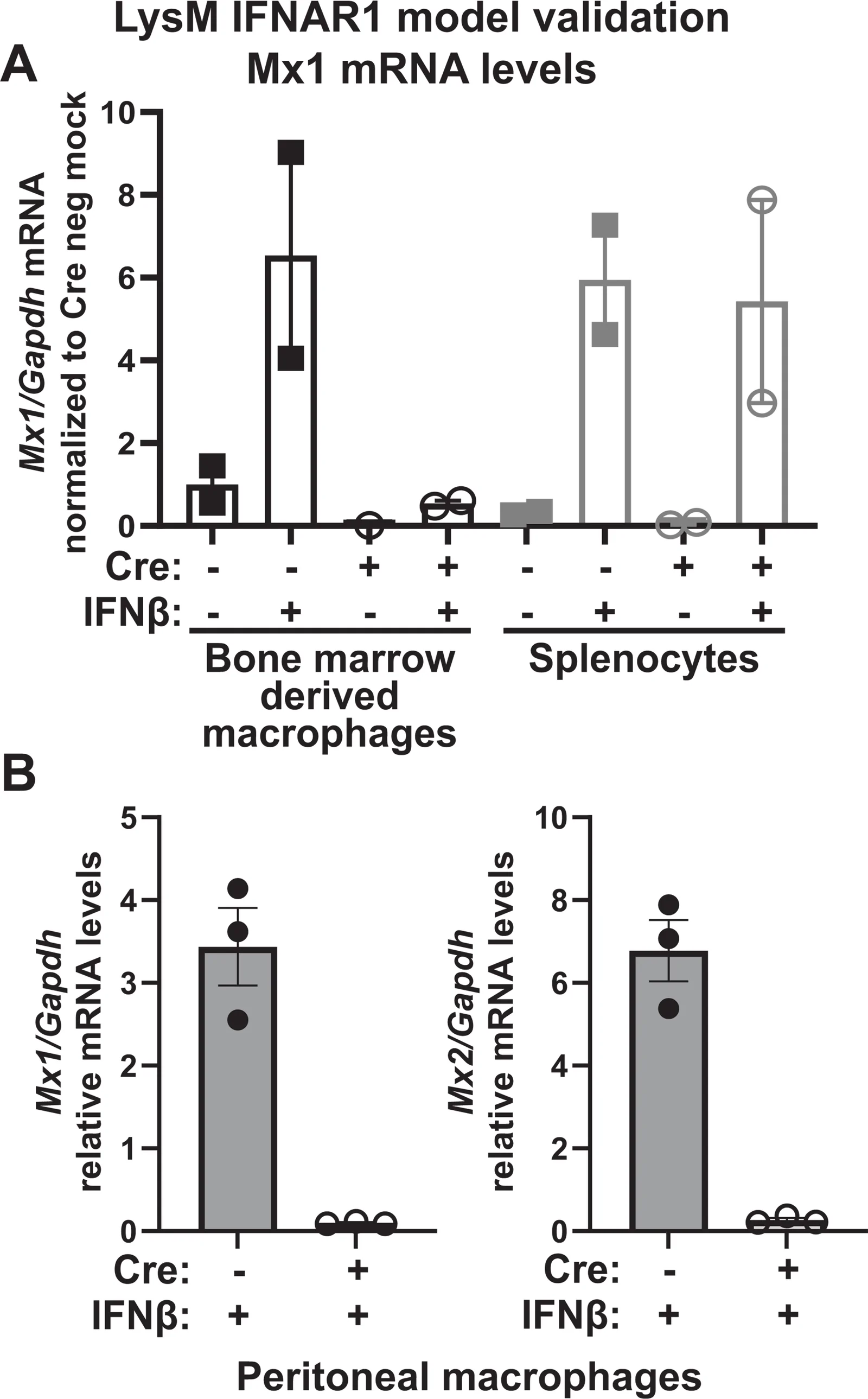
Validation of mouse model of myeloid cell-specific IFNAR1 deficiency. (**A**) Bone marrow-derived macrophages and total splenocytes were harvested from naïve *Lysm^Cre/wt^ Ifnar1^fl/fl^* and *Lysm^wt/wt^ Ifnar1^fl/fl^* littermates (referred to as LysM IFNAR1 Cre-positive and Cre-negative in subsequent figure legends). Cultures were treated with 10 U/mL recombinant IFNβ or carrier for 4 h. Total RNA was subjected to qRT-PCR to measure relative levels of *Mx1* mRNA normalized to corresponding relative *gapdh* mRNA levels. Data were subsequently normalized to relative levels of expression in the Cre-negative carrier-treated condition (set to 1). (**B**) Peritoneal cells were lavaged from naïve LysM IFNAR1 Cre-positive and Cre-negative littermates. Peritoneal macrophages (enriched using anti-F4/80 conjugated magnetic beads) were treated *ex vivo* with 10 U/mL recombinant IFNβ for 4 h, and relative mRNA levels of Mx1 and Mx2 were measured as above.

Primary bone marrow-derived macrophages from *Lyz2^wt/wt^Ifnar1^fl/fl^* and *Lyz2^Cre/wt^Ifnar1^fl/fl^* littermates (referred to as Cre-negative and LysM IFNAR1 Cre-positive, respectively, throughout the article) were used to validate the new mouse model generated by our group. Macrophages, along with splenocytes from the same donors, were treated *ex vivo* with IFNβ, and ISG expression was measured at 4 h post-treatment. As expected, IFNβ treatment increased *Mx1* mRNA levels in Cre-negative macrophages and splenocytes (Fig. 1A). mRNA levels of *Mx1* were similar in Cre-negative and LysM IFNAR1 Cre-positive splenocytes, consistent with only a small proportion of total splenocytes being represented by LysM-expressing myeloid cells. In contrast, mRNA levels of *Mx1* were only minimally increased in LysM IFNAR1 Cre-positive macrophages. To increase the stringency of validation, peritoneal macrophages enriched from Cre-negative and LysM IFNAR1 Cre-positive littermates were treated with IFNβ *ex vivo*, with subsequent measurement of *Mx1* and *Mx2* mRNA levels as above (Fig. 1B). Similar differential ISG expression was observed in bone marrow-derived macrophages and peritoneal macrophages tested directly *ex vivo*, validating the genetic model of myeloid cell-specific IFNAR1 deficiency (Fig. 1).

### Myeloid cell-specific IRF-3 or IFNAR1 deficiency does not affect acute replication of the orf36 null MHV68 mutant

**Fig 2:**
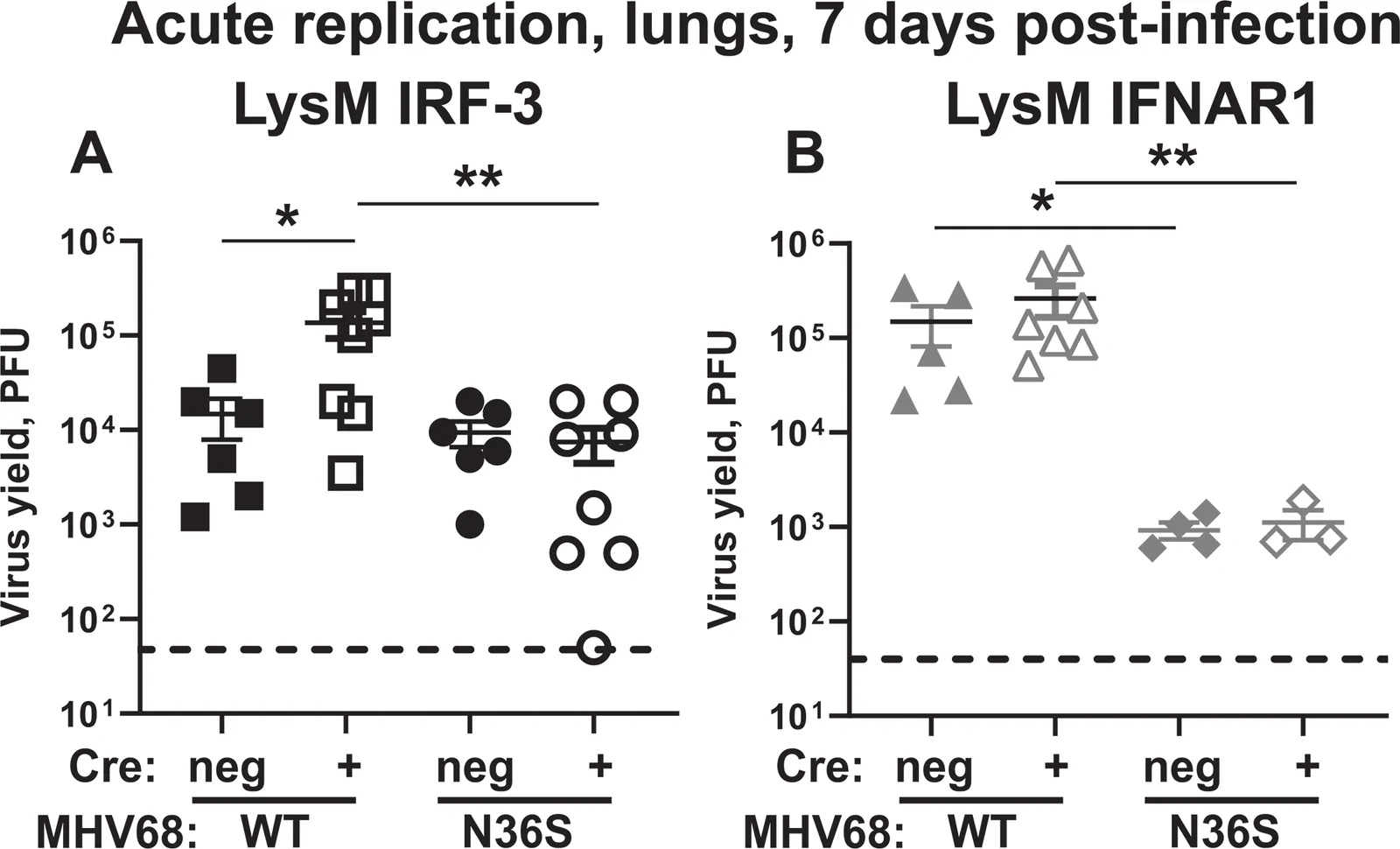
Myeloid cell-specific IRF-3 or IFNAR1 deficiency does not affect acute replication of the orf36 null MHV68 mutant. *Lysm^Cre/wt^Irf3^fl/fl^* and *Lysm^wt/wt^Irf3^fl/fl^* littermates (referred to as LysM IRF-3 Cre-positive and Cre-negative in subsequent figure legends) (**A**) or LysM IFNAR1 Cre-positive and Cre-negative mice (**B**) were inoculated intranasally with 10,000 plaque-forming units (PFU) of WT or orf36 null (N36S) MHV68. Lungs were harvested at 7 days post-infection. Viral titers from homogenized lungs were determined by plaque assay using a 3T12 fibroblast cell line. Each symbol represents an individual mouse. The mean and standard error of the mean are shown here and in subsequent figures. The dotted line represents the limit of detection. *P < 0.05; **P < 0.01. Here and in remaining figures, only statistically significant differences are noted in the figure panels.

To test the role of myeloid cell-intrinsic IRF-3 expression during acute MHV68 replication, *Lyz2^wt/wt^Irf3^fl/fl^* and *Lyz2^Cre/wt^Irf3^fl/fl^* littermates (referred to as Cre-negative and LysM IRF-3 Cre-positive throughout the article) were intranasally infected with 10,000 PFU of WT or N36S MHV68. Peak WT MHV68 lung titers were significantly increased in LysM IRF-3 Cre-positive as compared to Cre-negative littermates (Fig. 2A). N36S and WT MHV68 acute titers were similar in LysM Cre-negative lungs, in contrast to that observed in previous studies (16, 27). This is likely due to the effect of genetic background: C57BL/6J mice were used in our previously published studies, whereas the LysM-IRF-3 model generated by the Taniguchi group used embryonic stem cell line of mixed CBA and C57BL/6 genetic backgrounds (30, 32). Finally, unlike that observed for WT MHV68, myeloid cell-specific IRF-3 deficiency had no effect on the acute titers of the N36S MHV68 mutant (Fig. 2A).

Next, acute lung titers were examined in LysM IFNAR1 Cre-positive and Cre-negative littermates. The lack of myeloid cell-specific type I IFN signaling had no effect on the acute lung titers of WT MHV68 (Fig. 2B). Acute replication of the N36S mutant was attenuated, as compared to WT MHV68, in Cre-negative lungs (Fig. 2B). This was an expected outcome, as *Lyz2^Cre/wt^* and *Ifnar1^fl/fl^* mice used to generate this mouse model are on the C57BL/6J genetic background. Finally, myeloid cell-specific IFNAR1 deficiency had no effect on the N36S acute lung titers (Fig. 2B). In summary, myeloid cell-specific IRF-3, but not IFNAR1 deficiency, resulted in increased acute lung titers of WT MHV68, with neither affecting acute replication of the N36S MHV68 mutant.

### Myeloid cell-specific IFNAR1 or IRF-3 deficiency results in decreased persistent replication of wild-type MHV68 in the lungs

**Fig 3:**
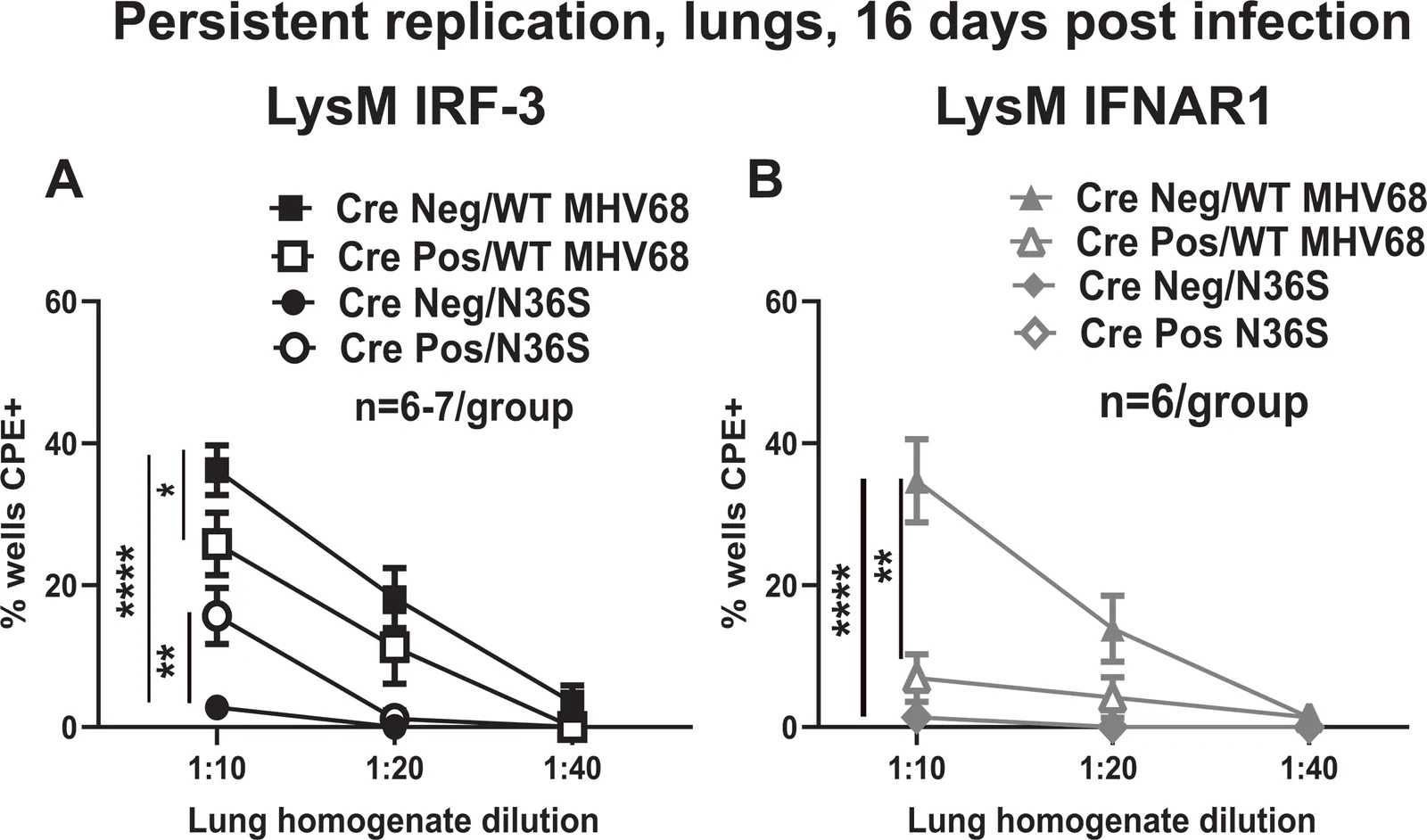
Myeloid cell-specific IFNAR1 or IRF-3 deficiency results in decreased persistent replication of wild-type MHV68 in the lungs. LysM IRF-3 Cre-positive and Cre-negative mice (**A**) or LysM IFNAR1 Cre-positive and Cre-negative mice (**B**) were infected as in Fig. 2. Lungs were harvested at 16 days post-infection, homogenized, and serial dilutions cultured on mouse embryonic fibroblast (MEF) monolayers (12 replicates/dilution). Cytopathic effect (CPE) was assessed after 14 days of incubation and graphed for each dilution as % wells positive for CPE. Data were pooled from the indicated number of animals within each group. Statistical analyses were performed using data from 1:10 lung homogenate dilution. *P < 0.05; **P < 0.01; ****P < 0.0001.

Acute MHV68 replication in the lungs is cleared by 10–12 days post-infection, as determined by the conventional plaque assay of lung homogenates using the 3T12 mouse fibroblast cell line (33). However, very low levels of persistent MHV68 replication are detectable in the lungs of wild-type mice for at least 30 days post-infection using a highly sensitive mouse embryonic fibroblast (MEF)-based semiquantitative assay (34). Using this assay, we showed increased persistent replication of WT MHV68 and N36S mutant in the lungs of mice with myeloid-specific STAT1 deficiency (27) and decreased persistent WT MHV68 replication in *Irf3^−/−^* lungs (16). Levels of persistent WT MHV68 replication were also decreased in mice with myeloid-specific IRF-3 deficiency, despite increased acute lung titers (Fig. 3A, acute lung titers shown in Fig. 2A). Persistent replication of the N36S mutant was attenuated, as compared to WT MHV68, in Cre-negative lungs (Fig. 3A). In contrast to that observed for WT MHV68, an increase in persistent N36S replication was observed in LysM IRF-3 Cre-positive as compared to Cre-negative lungs (Fig. 3A).

*Ifnar1^−/−^* mice display increased acute MHV68 replication in several organs, including lungs, for up to 3 weeks post-infection (14, 15). Surprisingly, levels of persistent WT MHV68 replication were decreased in lungs of mice with myeloid-specific IFNAR1 deficiency at 16 days post-infection (Fig. 3B). Attenuated persistent replication of the N36S mutant was not modified by the myeloid cell-specific IFNAR1 deficiency (Fig. 3B). In summary, myeloid cell-specific IFNAR1 and, to a lesser extent, IRF-3 deficiency led to attenuated persistent lung replication of WT MHV68, revealing a proviral role of myeloid-intrinsic type I IFN signaling and IRF-3 expression in supporting persistent MHV68 replication in the lungs during chronic gammaherpesvirus infection.

### Myeloid-specific IFNAR1 but not IRF-3 deficiency leads to rescue of the latent reservoir and *ex vivo* reactivation of the N36S MHV68 mutant in the chronically infected spleen

Following transition through LysM-expressing cells, MHV68 establishes a latent reservoir in splenic B cells (25), with the peak latent reservoir observed at 16 days post-infection. Optimal establishment of a latent reservoir in splenic B cells requires expression and enzymatic activity of MHV68 orf36 (11). Importantly, myeloid-specific STAT1 deficiency rescues the establishment of the N36S latent reservoir in splenic B cells, but not viral reactivation (27).

**Fig 4:**
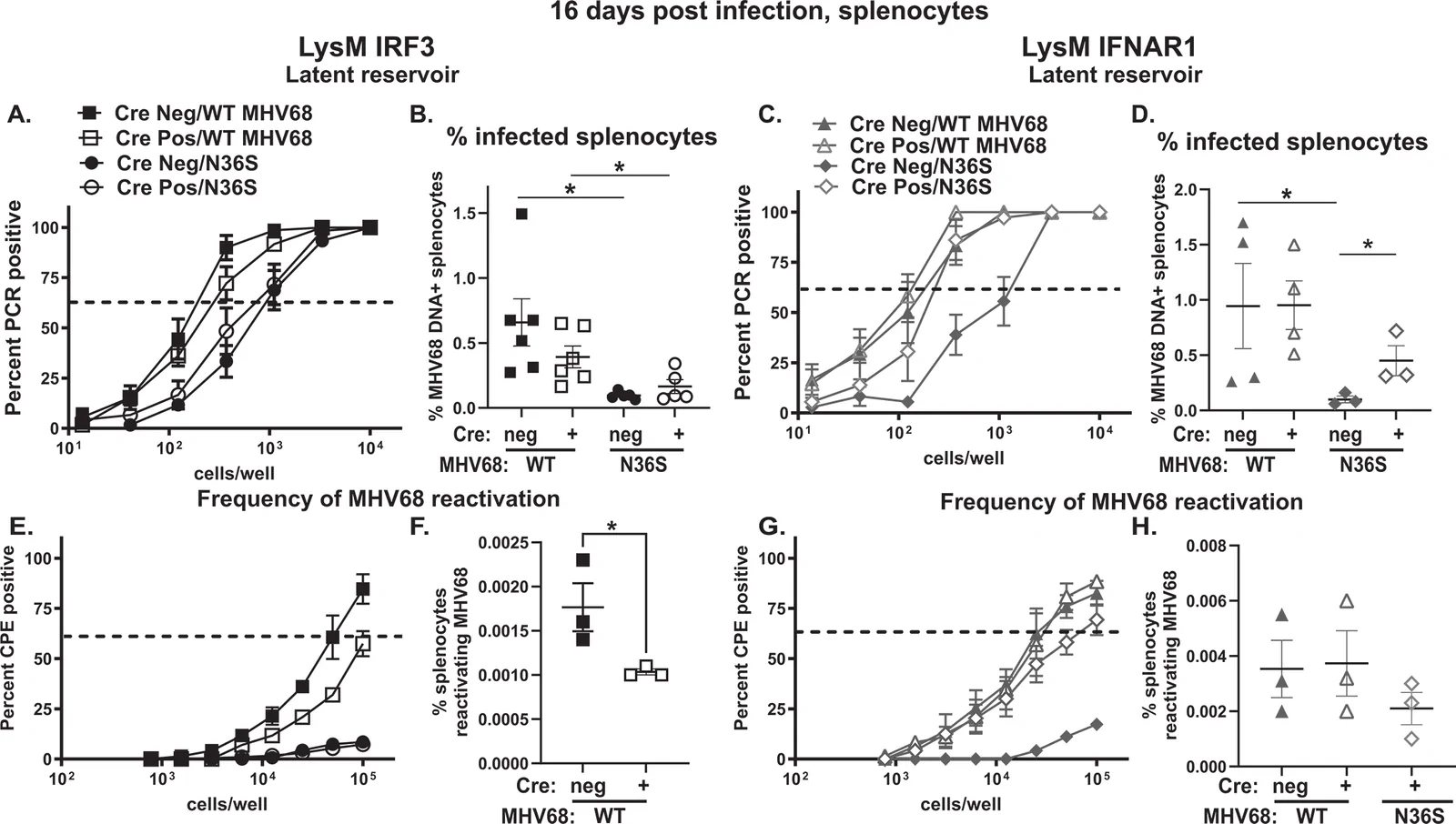
Myeloid-specific IFNAR1 but not IRF-3 deficiency leads to rescue of the latent reservoir and *ex vivo* reactivation of the N36S MHV68 mutant in the chronically infected spleen. LysM IRF-3 Cre-positive and Cre-negative (**A, B, E, and F**) or LysM IFNAR1 Cre-positive and Cre-negative littermates (**C, D, G, and H**) were infected as in Fig. 2. Splenocytes were pooled within each experimental group (three to four mice per group per experiment) and subjected to limiting dilution assays at 16 days post-infection. (**A and C**) The frequency of MHV68 DNA-positive splenocytes was determined by limiting dilution PCR. The intersection of the sigmoidal curve and the dotted line drawn at *Y* = 63.2% indicates the inverse frequency of a positive event as defined by the corresponding *x* coordinate. Pooled data are presented. In panels **B **and **D**, inverse frequency of MHV68 DNA-positive splenocytes was converted to percent MHV68 DNA^+^ splenocytes, with each symbol representing data obtained from pooled spleens collected in an independent study. (**E and G**) The frequency of MHV68 *ex vivo* reactivation was determined by limiting dilution assay. Pooled data are presented. In panels **F **and **H**, inverse frequency of *ex vivo* reactivation from splenocytes was converted to percent splenocytes reactivating MHV68 *ex vivo*. Each symbol represents data obtained from pooled spleens collected in an independent study. Only values from experimental groups where quantitation was possible (based on the detection of intersection of the sigmoidal curve and the dotted line drawn at *Y* = 63.2%) are presented in panels **F **and **H**. *P < 0.05.

Having examined acute and persistent lung replication of WT and N36S MHV68, the effect of myeloid cell-specific IRF-3 or IFNAR1 deficiency was assessed during the establishment of chronic infection. Myeloid cell-specific IRF-3 deficiency did not affect the establishment of WT MHV68 latent reservoir in the spleen, as determined by measuring the frequency and percentage of MHV68 DNA^+^ splenocytes (Fig. 4A and B). As expected, the frequency and percentage of MHV68 DNA^+^ splenocytes were decreased in Cre-negative mice infected with N36S mutant as compared to WT MHV68 (Fig. 4A and B). The frequency and percentage of MHV68 DNA^+^ splenocytes remained attenuated in N36S-infected as compared to WT MHV68-infected LysM IRF-3 Cre-positive spleens (Fig. 4A and B).

Similar to that observed for IRF-3, myeloid cell-specific IFNAR1 deficiency did not affect the frequency and percent of splenocytes infected with WT MHV68 (Fig. 4C and D). In contrast, lack of IFNAR1 in myeloid cells led to an increase in the splenic reservoir of the N36S mutant, with no statistically significant difference in percentage of infected splenocytes observed between WT- and N36S-infected LysM IFNAR1 Cre-positive mice (Fig. 4C and D). This rescue of the N36S latent reservoir in LysM IFNAR1 Cre-positive mice occurred despite similarly attenuated acute N36S lung titers in LysM IFNAR1 Cre-positive and Cre-negative mice (Fig. 2B).

While the MHV68 splenic latent reservoir is largely supported by germinal center B cells at 16 days post-infection, MHV68 and Epstein-Barr virus (EBV) reactivation from B cells is primarily driven by the differentiation of infected B cells into plasma cells (35, 36). Therefore, *ex vivo* reactivation of WT and N36S MHV68 from splenocytes was assessed next. Interestingly, despite no difference in the splenic latent reservoir, the frequency and percentage of *ex vivo* reactivation of WT MHV68 were significantly decreased in splenocytes of chronically infected mice with myeloid cell-specific IRF-3 deficiency (Fig. 4E and F). It was not technically possible to determine if similar attenuation also occurred in N36S-infected splenocytes due to very low levels of *ex vivo* reactivation (Fig. 4E).

A similar frequency and percentage of *ex vivo* reactivation from splenocytes were observed in WT MHV68-infected Cre-negative and LysM IFNAR1 Cre-positive littermates (Fig. 4G and H). However, the attenuated *ex vivo* reactivation of the N36S mutant was rescued in splenocytes of chronically infected mice with myeloid-specific IFNAR1 deficiency (Fig. 4G and H). In summary, myeloid cell-specific IRF-3 deficiency led to decreased *ex vivo* reactivation of WT MHV68 from splenocytes without affecting the latent splenic viral reservoir of WT or N36S MHV68. Myeloid cell-specific IFNAR1 deficiency did not affect either the splenic latent reservoir or *ex vivo* reactivation of WT MHV68. In contrast, the attenuated establishment of latent splenic reservoir and *ex vivo* reactivation of the N36S mutant were rescued by the myeloid-specific IFNAR1 deficiency.

### Attenuated germinal center response driven by N36S MHV68 mutant is not rescued by myeloid cell-specific IRF-3 or IFNAR1 deficiency

Establishment of the splenic latent reservoir is intimately tied to manipulation of B cell differentiation by gammaherpesviruses. Specifically, EBV and MHV68 infect naïve B cells, switch to latent life cycle, and drive differentiation of infected and bystander B cells through the germinal center response (37–39). Robust proliferation of latently infected germinal center B cells leads to an exponential increase in the splenic latent reservoir; differentiation of infected germinal center B cells into plasma cells supports viral reactivation (35, 36). Similar to that observed during physiological germinal center responses, MHV68-driven germinal center responses require interaction of B cells with CD4^+^ T follicular helper cells (40, 41).

We previously showed that expression and enzymatic activity of orf36 is required for optimal MHV68-driven germinal center responses during chronic infection (11). Interestingly, expression of KSHV-encoded orf36 was sufficient to drive germinal center B cell differentiation and, eventually, B cell lymphomas in uninfected KSHV orf36 transgenic mice, underscoring the important and conserved role of gammaherpesvirus protein kinases in viral manipulation of B cells (42). Having observed a rescue of the latent reservoir and *ex vivo* reactivation of the N36S mutant in the spleens of mice with myeloid cell-specific IFNAR1, but not IRF-3 deficiency, germinal center responses were assessed next.

**Fig 5:**
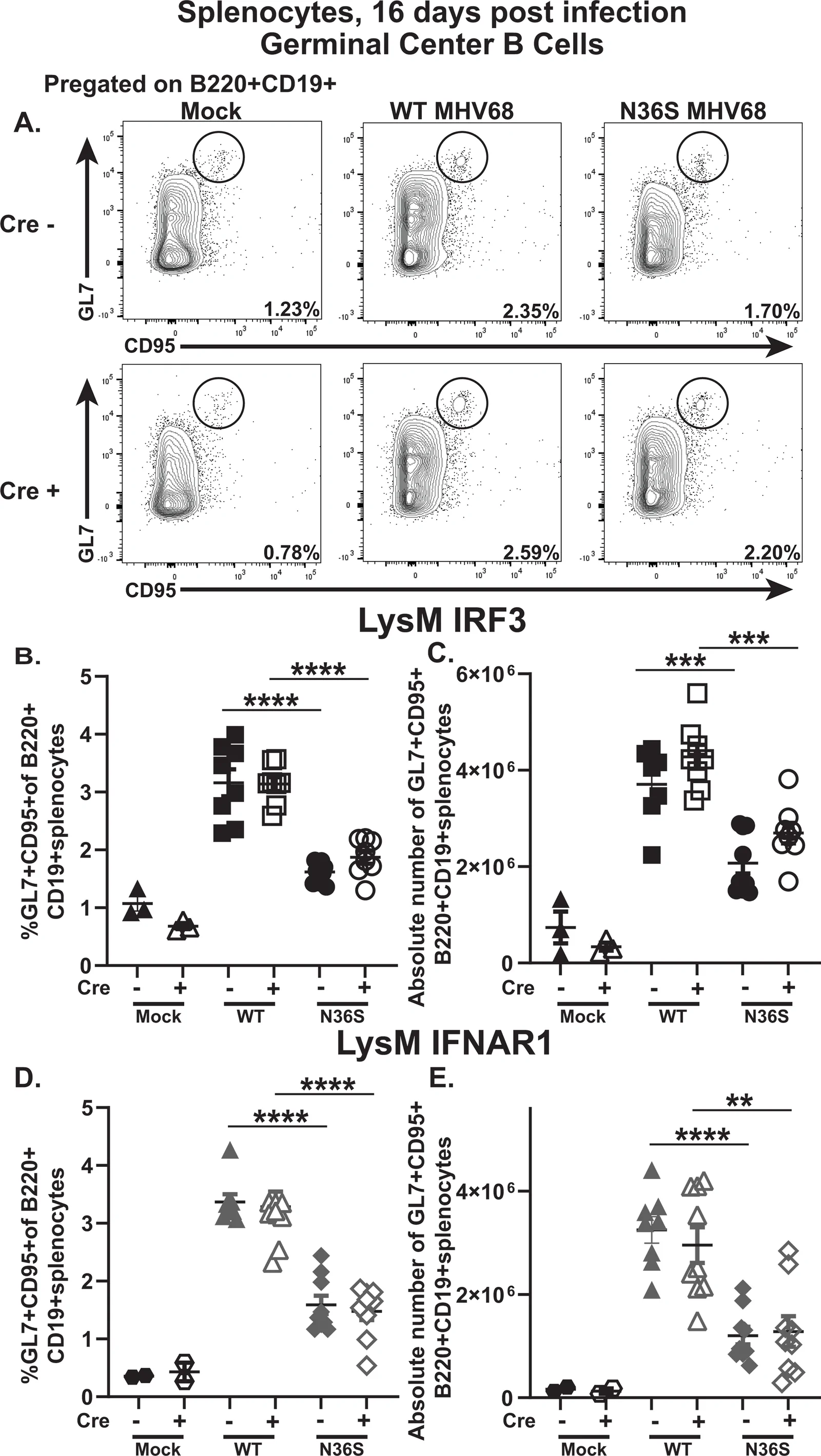
Attenuated abundance of germinal center B cells following N36S MHV68 mutant infection is not rescued by myeloid cell-specific IRF-3 or IFNAR1 deficiency. (**A**) Representative flow cytometry gating strategy for germinal center B cells defined as CD19^+^B220^+^GL7^+^CD95^+^. LysM IRF-3 Cre-positive and Cre-negative (**B and C**) or LysM IFNAR1 Cre-positive and Cre-negative littermates (**D and E**) were mock-infected or infected as in Fig. 2. Splenocytes from individual mice were analyzed at 16 days post infection. The frequency (**B**) and absolute number (**C**) of germinal center B cells from mock, wild type, or N36S MHV68-infected LysM IRF-3 Cre-positive and Cre-negative mice. The frequency (**D**) and absolute number (**E**) of germinal center B cells from mock, wild-type, or N36S MHV68-infected LysM IFNAR1 Cre-positive and Cre-negative mice. Each symbol represents an individual spleen. **P < 0.01; ***P < 0.001; ****P < 0.0001.

The frequency and absolute numbers of germinal center B cells, defined using the gating strategy in Fig. 5A, were not affected by myeloid-specific IRF-3 or IFNAR1 deficiency in WT MHV68-infected mice (Fig. 5B through E). As expected, attenuated frequency and absolute numbers of germinal center B cells were observed in N36S-infected as compared to WT MHV68-infected Cre-negative mice across all studies (Fig. 5B through E). Myeloid cell-specific IRF-3 deficiency did not affect the attenuated germinal center B cell populations during N36S mutant infection (Fig. 5B and C). Interestingly, despite rescue of the splenic latent reservoir and *ex vivo* reactivation of the N36S mutant, germinal center B cell frequency and abundance remained attenuated in mice with myeloid cell-specific IFNAR1 deficiency (Fig. 5D and E).

**Fig 6:**
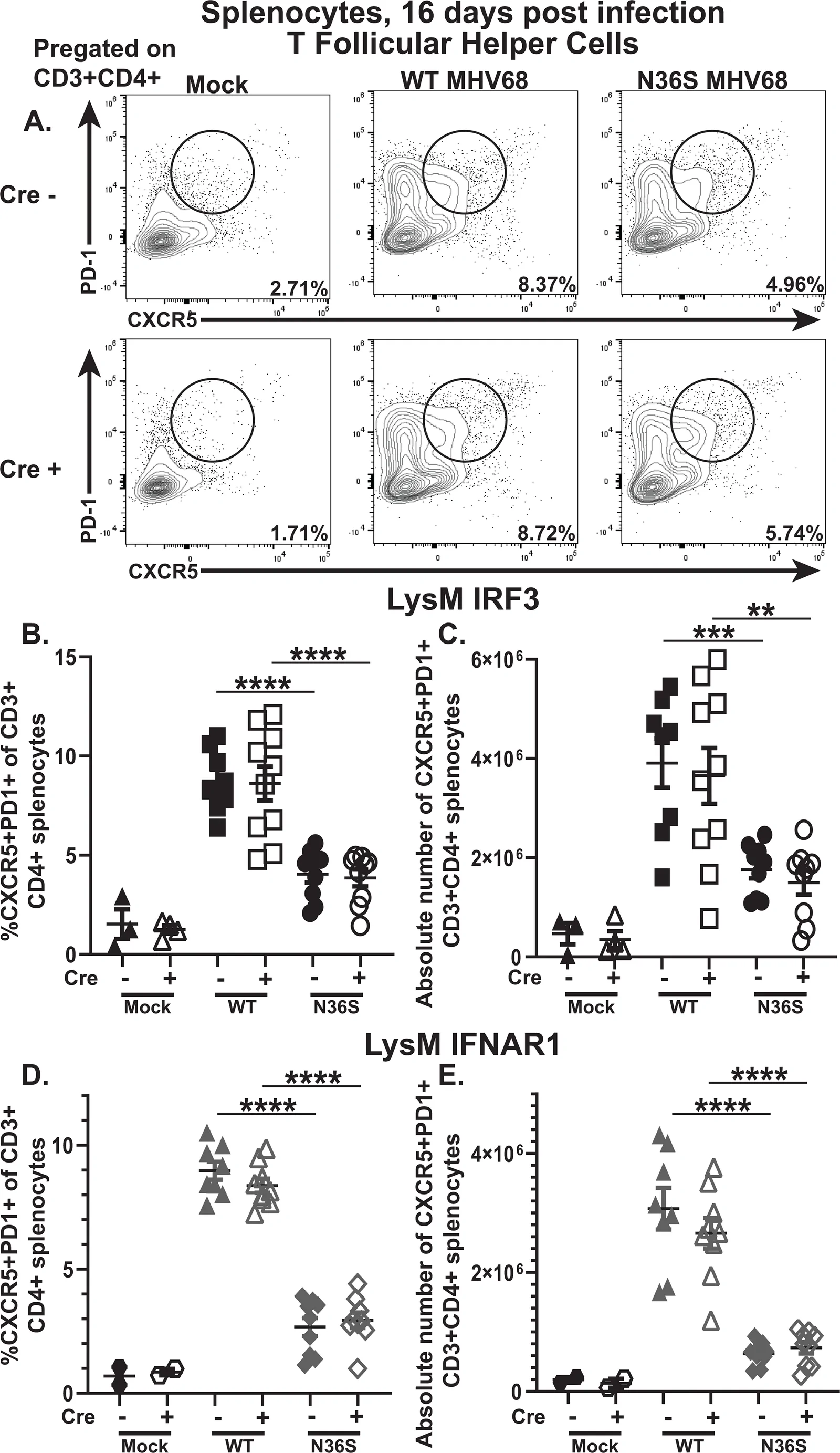
Attenuated abundance of T follicular helper cells following N36S MHV68 mutant infection is not rescued by myeloid cell-specific IRF-3 or IFNAR1 deficiency. (**A**) Representative flow cytometry gating strategy for T follicular helper cells defined as CD3^+^CD4^+^CXCR5^+^PD-1^+^. LysM IRF-3 Cre-positive and Cre-negative (**B and C**) or LysM IFNAR1 Cre-positive and Cre-negative littermates (**D and E**) were mock-infected or infected as in Fig. 2. Splenocytes from individual mice were analyzed at 16 days post-infection. The frequency (**B**) and absolute number (**C**) of T follicular helper cells from mock, wild type, or N36S MHV68-infected LysM IRF-3 Cre-positive and Cre-negative mice. The frequency (**D**) and absolute number (**E**) of T follicular helper cells from mock, wild type, or N36S MHV68-infected LysM IFNAR1 Cre-positive and Cre-negative mice. Each symbol represents an individual spleen. **P < 0.01; ***P < 0.001; ****P < 0.0001.

CD4 T follicular helper cells are critical for physiological and MHV68-driven germinal center responses (40, 41). Due to positive feedback between germinal center B cells and T follicular helper cells, changes in germinal center B cell population are usually (but not always) mirrored by corresponding changes in the T follicular helper cell population, including during chronic MHV68 infection. Indeed, frequency and absolute numbers of CD4 T follicular helper cells remained decreased in N36S-infected as compared to WT MHV68-infected mice regardless of expression of IRF-3 or IFNAR1 by myeloid cells (Fig. 6B through E, gating strategy in panel A). In summary, despite rescued N36S latent reservoir and *ex vivo* reactivation in splenocytes of mice with myeloid cell-specific IFNAR1 but not IRF-3 deficiency, N36S-driven germinal center responses remained attenuated in both mouse models.

## DISCUSSION

Our previous study had taken advantage of a combination of host and viral genetics to demonstrate that myeloid cell-intrinsic antagonism between STAT1 and conserved gammaherpesvirus protein kinase ensures optimal establishment of the viral latent reservoir in splenic B cells during chronic infection (27). In addition to its role as a critical effector of IFN responses, STAT1 is also activated by non-IFN cytokines. Thus, the current study aimed to define the extent to which the previously observed viral protein kinase-STAT1 antagonism in the context of LysM-expressing cells reflected antagonism of type I IFN responses. Specifically, this study focused on IRF-3, a well-established inducer of type I IFN expression, and type I IFN signaling mediated by the cognate receptor. We found that many viral and host phenotypes associated with myeloid cell-specific IFNAR1 deficiency phenocopied those observed in myeloid cell-specific STAT1 deficiency during chronic infection (viral and host phenotypes summarized in Table 1). In contrast, attenuated WT MHV68 reactivation observed in mice with myeloid cell-specific IRF-3 deficiency offered a mechanistic insight into the proviral phenotypes of global IRF-3 expression observed in our previous study (16).

**TABLE 1:** Summary of host and viral phenotypes^*a*^

Viral and host phenotype	Myeloid cell-specific IRF-3 deficiency	Global IRF-3 deficiency (16, 43)	Myeloid cell-specific IFNAR1 deficiency	Myeloid cell-specific STAT1 deficiency (27)
Acute lung replication	WT MHV68 titers increased; N36S titers not affected	WT MHV68 titers increased; N36S titers not affected	No effect on either WT or N36S MHV68 titers	WT MHV68 titers increased; N36S titers not affected
Persistent lung replication	Decreased for WT MHV68; increased for N36S mutant	Decreased for WT MHV68 (1,000 PFU inoculum dose)	Decreased for WT MHV68	Increased for WT and N36S MHV68
Splenic latent reservoir	Not affected for WT MHV68 or N36S mutant	Decreased for WT MHV68 (1,000 PFU inoculum dose); not affected for WT MHV68 or N36S mutant (10,000 PFU inoculum dose)	Not affected for WT MHV68; N36S latent reservoir is rescued	Not affected for WT MHV68; N36S latent reservoir is rescued
*Ex vivo* reactivation from splenocytes	Decreased for WT MHV68	Not affected for WT MHV68 or N36S mutant	Not affected for WT MHV68; reactivation of N36S mutant is rescued	Not affected for WT MHV68 or the N36S mutant
Germinal center responses (germinal center B cells and T follicular helper cells)	Not affected in WT MHV68 infection; remain attenuated in N36S infection	Germinal center B cells not affected in WT MHV68 infection and remain attenuated in N36S infection; proportion of T follicular helper cells increased in WT- and N36S-infected *Irf3^−/−^* mice	Not affected in WT MHV68 infection; remain attenuated in N36S infection	Not affected in WT MHV68 infection; remain attenuated in N36S infection

^*a*^ WT MHV68 infection of *Irf3^−/−^* mice in Johnson et al.’s study (16) was examined using two inoculum doses (1,000 PFU and 10,000 PFU), with only a single N36S inoculum dose (10,000 PFU). The current study and the published study of myeloid cell-specific STAT1 deficiency (27) exclusively used the 10,000 PFU inoculum dose for WT and N36S MHV68.

### Role of myeloid cell-intrinsic IRF-3 expression during natural gammaherpesvirus infection

We previously demonstrated that IRF-3 expression was critical for induction of type I IFN and attenuation of WT MHV68 replication in primary bone marrow-derived macrophages (12). Accordingly, attenuated replication of the N36S MHV68 mutant was partially rescued in *Irf3^−/−^* macrophages (4, 12). Consistent with the requirement of IRF-3 for type I IFN expression, acute lung titers of WT MHV68 were increased in *Irf3^−/−^* mice (16, 43). In contrast, acute lung replication of the N36S MHV68 mutant was not affected by the global IRF-3 deficiency (16). In the current study, myeloid cell-specific IRF-3 deficiency led to increased acute lung titers of WT MHV68, but not the N36S mutant (Fig. 1B), suggesting that LysM-expressing myeloid cells in the lungs contribute to the expression of type I IFN to control acute MHV68 replication in several lung cell types. However, it is intriguing that myeloid cell-specific IFNAR1 deficiency did not lead to an increase in WT MHV68 titers (Fig. 2B; also see discussion below). The lack of congruence between IRF-3-dependent and IFNAR1-dependent acute replication phenotypes for WT MHV68 suggests that IRF-3 might have additional antiviral IFN-independent functions that restrict MHV68 replication, a possibility to be tested in future studies. In contrast to that observed for WT MHV68, myeloid-specific IRF-3 deficiency failed to increase acute lung titers of the N36S mutant (Fig. 2A). Because MHV68 orf36 promotes multiple steps of MHV68 replication, this outcome indicates that IRF-3 deficiency is not sufficient to fully rescue N36S replication in lungs or, as we observed, in primary macrophages (9, 12).

We previously showed that despite increased acute MHV68 lung titers, chronically infected *Irf3^−/−^* mice showed decreased persistent WT MHV68 replication in the lungs at 16 days post-infection (16). In the current study, WT MHV68 persistent lung replication was decreased in mice with myeloid cell-specific IRF-3 deficiency (Fig. 3A), suggesting that myeloid cell-specific IRF-3 expression may contribute to the previously observed proviral phenotype of global IRF-3 expression.

Despite the reported attenuation of IRF-3 transcriptional activity by the conserved gammaherpesvirus kinases, including MHV68 orf36 (4), the attenuated latent reservoir and *ex vivo* reactivation of the N36S MHV68 mutant were not rescued in the chronically infected *Irf3^−/−^* mice (16). Correspondingly, as observed in the current study, myeloid-specific IRF-3 deficiency had very little, if any, effect on the attenuated N36S latent reservoir or reactivation in the spleen (Fig. 4, Table 1). However, despite a similar magnitude of the latent reservoir, the frequency and proportion of splenocytes supporting *ex vivo* reactivation of WT MHV68 were decreased in mice with myeloid-specific IRF-3 deficiency (Fig. 4E and F).

### Role of myeloid cell-intrinsic IFNAR1 expression during natural gammaherpesvirus infection

A significant increase in acute and persistent MHV68 replication is observed in *Ifnar1^−/−^* mice for up to 4 weeks post-infection, along with increased MHV68 reactivation during long-term chronic infection (14, 15, 44). However, myeloid cell-specific IFNAR1 deficiency did not affect acute titers of either WT or the N36S MHV68 in the current study (Fig. 1B and C). A majority of infected lung cells are represented by epithelial cells that can respond to all three types of IFN in both Cre-negative and LysM IFNAR1 Cre-positive mice (24). Furthermore, expression of type I IFN is increased in MHV68-infected *Ifnar1^−/−^* bone marrow-derived macrophages as compared to control-infected macrophages *in vitro* (12). Thus, a potential increase in type I IFN expression in LysM IFNAR1 Cre-positive lungs may have further attenuated MHV68 replication in epithelial cells to offset a presumed increase in replication in LysM-expressing IFNAR1-deficient macrophages.

Unexpectedly, persistent lung replication of WT MHV68 was attenuated in mice lacking IFNAR1 expression in myeloid cells. Following clearance of acute replication in the lungs, including acutely infected epithelial cells, MHV68 shifts its tropism to lung B cells, with a similar frequency of infection observed in CD19^+^ B cells and CD19^−^ lung cells at 16 days post-infection (45). Global IFNAR1 deficiency leads to dysfunctional MHV68-specific CD8 T cell response that contributes to uncontrolled MHV68 chronic infection (46). In contrast, myeloid cell-specific IFNAR1 deficiency is not expected to have any effect on differentiation and function of antiviral CD8 T cells, similar to what we showed for myeloid cell-specific STAT1 deficiency (27). Thus, it is possible that increased expression of lytic MHV68 genes in IFNAR1- or IRF-3 deficient lung macrophages increased susceptibility of these cells to detection and elimination by antiviral CD8 T cells at 16 days post-infection.

Similar to that previously observed for myeloid cell-specific STAT1 deficiency, lack of IFNAR1 expression by myeloid cells resulted in rescue of the attenuated splenic latent reservoir of the N36S MHV68 mutant (Fig. 4C and D). This observation suggests that the conserved protein kinases antagonize myeloid cell-specific type I IFN signaling, but not expression, to promote establishment of chronic infection in the spleen.

Interestingly, and in contrast to that observed in mice with myeloid cell-specific STAT1 deficiency, the frequency of *ex vivo* N36S mutant reactivation was rescued in mice with IFNAR1-deficient myeloid cells. Differentiation of infected germinal center or memory B cells into plasma cells is thought to primarily support reactivation of EBV and MHV68 (35, 36). However, the observed rescue of N36S splenic reactivation in LysM IFNAR1 mice (Fig. 4G) and attenuated reactivation of WT MHV68 in mice with myeloid cell-specific IRF-3 deficiency (Fig. 4E) suggests that LysM-expressing myeloid cells and their IFN signaling/IRF-3 expression status may affect differentiation of infected B cells into plasma cells. Interestingly, follicular dendritic cells that support of germinal center responses can express IFNα, drive germinal center differentiation of self-reactive B cells, and promote pathogenic antibody expression in lupus mouse models (47). However, follicular dendritic cells do not express Lysozyme M and are not affected in the mouse models used in the current study, as evidenced by the lack of Cre-dependent germinal center phenotypes (Fig. 5 and 6). Interestingly, targeting of antigen to splenic macrophages using the anti-CD169-antigen conjugates resulted in robust stimulation of antigen-specific germinal center responses and antibody titers (48). Thus, future studies are needed to define the mechanisms underlying viral reactivation phenotypes observed in the current study.

## MATERIALS AND METHODS

### Animal studies

*Lys2^Cre/wt^* (26) and *Ifnar1^fl/fl^* (31) mice were purchased from Jackson Laboratories (Bar Harbor, ME, USA). *Lys2^Cre/wt^Irf3^fl/fl^* mice were a kind gift of Dr. Taniguchi (30). Mouse model of myeloid cell-specific IFNAR1 deficiency was generated by crossing *Lys2^Cre/wt^* mice and *Ifnar1^fl/fl^* mice to produce *Lys2^Cre/wt^ Ifnar1^fl/fl^* and *Lys2^wt/wt^ Ifnar1^fl/fl^* breeders. All mouse strains used for experiments were bred and housed in a pathogen-free facility at MCW. At 6–7 weeks of age, Cre-positive and Cre-negative littermates within each strain were intranasally infected under light anesthesia with 10,000 plaque-forming units (PFU) of corresponding virus in 15 μL of sterile, serum-free Dulbecco’s modified Eagle’s medium (DMEM). Infections with the N36S MHV68 mutant were controlled by the parental virus retaining a single LoxP site (referred to as wild type in the figures and text) (4). The MHV68 stocks were prepared and titered using the NIH 3T12 fibroblast cell line.

### Limiting dilution assays

The frequency of MHV68 DNA-positive cells was determined as previously described (49). In brief, in each independent experiment, splenocytes were pooled from all mice in each respective experimental group (3–4 mice/group), and 6, 3-fold, serial dilutions were subjected to a nested PCR reaction (12 replicates/dilution) using primers against the MHV68 genome. To determine the frequency of *ex vivo* reactivation, splenocytes were pooled from all mice in each respective experimental group (3–4 mice/group), and 8, 2-fold, serial dilutions of the cellular suspensions were plated onto a confluent monolayer of mouse embryonic fibroblasts (MEFs) in 24 replicates/dilution and cultured for 21 days. Viral reactivation in individual replicates for each dilution was scored positive or negative for cytopathic clearing of MEFs (performed by visual observation of the viral lysis of MEF monolayer) on day 21 of culture. Persistent MHV68 replication was measured in homogenized lungs (Magnalyzer, Roche) using 1 mm sterile zirconia beads and plated at 1:10, 1:20, and 1:40 dilutions on MEFs (12 replicates/dilution). Cytopathic clearing of MEFs was scored at 14 days of culture in each replicate as described above.

### Flow cytometry

Single-cell suspensions of splenocytes were prepared in fluorescence-activated cell sorting buffer (phosphate-buffered saline, 2% fetal bovine serum [FBS]). Cells (2 × 10^6^) were incubated with optimized antibody concentrations. Data were acquired using the Celesta (BD Biosciences, Franklin Lakes, NJ, USA) or Aurora (Cytek Biosciences, Fremont, California) flow cytometers (and data were analyzed using FlowJo software [BD Life Sciences, Franklin Lakes, NJ, USA]). The following antibodies used in this study were purchased from BioLegend (San Diego, CA, USA): B220-PECy7 (cat. 103222), CD19-PB (cat. 115523), GL7-APC (cat. 144617), GL7-FITC (cat. 144604), CD95-APC (cat. 152604), CD3-PB (cat. 100214), CD4-FITC (cat. 100406), PD-1-BV605 (cat. 135220), CXCR5-PE/Dazzle 594 (cat. 145522), B220-Spark Blue 574 (cat. 103289), CD19-BV510 (cat. 115545), CD3-Spark UV 387 (cat. 100283), CD4-BV510 (cat. 100553), and CXCR5-FITC (cat. 100553). CD95-PE-CF594 (cat. 562395) was purchased from BD Biosciences (Franklin Lakes, NJ, USA).

### Analyses of ISG expression: bone marrow-derived macrophages

Macrophages were derived from bone marrow flushed from femurs and tibias of littermates (5−6 weeks old) and cultured in DMEM containing 10% fetal bovine serum, 20% conditioned media derived from L929 cell line (as a source of M-CSF, ATCC), and 5% horse serum. Following 7 days of culture, adherent cells were lifted with versine (Thermo Fisher) and replated in DMEM containing 10% FBS, 10% L929-conditioned media, and 5% horse serum. On day 10, cultures were treated with 10U/mL recombinant mouse interferon beta (IFNβ) (BioLegend, San Diego, CA, USA) or carrier control. After 4 h of treatment, RNA was isolated and subjected to DNAse treatment, reverse transcription, and quantitative PCR. Relative expression was quantified by the delta-delta method using *Gapdh* as a housekeeping gene. The following primer pairs were used: *Gapdh* Forward, 5′-TGTGATGGGTGTGAACCACGAGAA-3′; *Gapdh* Reverse, 5′-GAGCCCTTCCACAATGCCAAAGTT-3′; *Mx1* Forward, 5′-AGCTAGACAGAGCAAACCAAGCCA-3′; *Mx1* Reverse, 5′-TCCCTGAAGCAGACACAGCTGAAA-3′; *Mx2* Forward, 5′-AGCAGAGTGACACAAGCGAGAAGA-3′; *Mx2* Reverse, 5′-AGCCCTTCTGTCCCTGAATCACAA-3′.

### Analyses of ISG expression: peritoneal macrophages

Peritoneal cells were pooled from 8-week-old naïve mice within each genotype (LysM IFNAR1 Cre-negative or Cre-positive; six mice/genotype). Peritoneal macrophages were isolated using magnetic beads provided within the EasySep Mouse F4/80 Positive Selection Kit (Cat. #100-0617, Stemcell Technologies, Vancouver, CA) according to the manufacturer’s instructions. In sum, peritoneal cells were suspended in sorting media (phosphate-buffered saline, 2% fetal bovine serum [FBS], and 1 mM EDTA) at 2.5 × 10^7^ cells/ mL. FcR-PolyBlock (50 µL/mL) and selection cocktail (25 µL/mL) were added to the cell suspension, followed by a 5-min incubation at room temperature. Next, 60 µL/mL of RapidSpheres were added to the cell suspension and incubated for 5-min at room temperature. After, the suspension volume was increased to 2.5 mL; the sample was placed into the EasySep magnet for 5 min. The cells mobilized to the tube wall by the magnet were enriched macrophages (F4/80-positive). This enrichment was performed three times. Next, the cells were resuspended in fresh media (DMEM, 10% FBS, 0.25% penicillin-streptomycin), plated, and allowed to rest for 2 h at 37°C before the addition of recombinant mouse IFNβ (10 U/mL) (BioLegend, San Diego, CA, USA) for 4 h at 37°C, with subsequent RNA extraction and quantitative reverse transcription-PCR (qRT-PCR) analyses as above. Samples were prepared in triplicate.

### Plaque assay

To determine viral titers in lungs from infected mice, lungs were harvested and homogenized (Magnalyzer, Roche) using 1 mm sterile zirconia beads. Serial 10-fold dilutions of lung homogenates were plated onto a subconfluent monolayer of 3T12 fibroblast cells and cultured for 7 days in the presence of methylcellulose in the overlay to prevent viral diffusion. Plaques were visualized by staining with neutral red.

### Statistical analyses

Statistical analyses were performed using a parametric *t*-test or non-parametric Mann-Whitney test. Comparisons assessed statistical significance of differences for a single virus (WT or N36S MHV68) across both host genotypes (Cre-negative and Cre-positive littermates) or differences between the two viruses within a single host genotype. Prism (GraphPad Software, Inc.) was used for all statistical analyses.

## Data Availability

All data associated with the study are available directly in the article.
